# What we talk about when we talk about medical librarianship: an analysis of Medical Library Association annual meeting abstracts, 2001–2019

**DOI:** 10.5195/jmla.2020.836

**Published:** 2020-07-01

**Authors:** Bethany Myers

**Affiliations:** 1 bethanymyers@library.ucla.edu, Louise M. Darling Biomedical Library, University of California Los Angeles (UCLA), Los Angeles, CA

## Abstract

**Objective:**

This study seeks to gain initial insight into what is talked about and whose voices are heard at Medical Library Association (MLA) annual meetings.

**Methods:**

Meeting abstracts were downloaded from the MLA website and converted to comma-separated values (CSV) format. Descriptive analysis in Python identified the number of presentations, disambiguated authors, author collaboration, institutional affiliation type, and geographic affiliation. Topics were generated using Mallet's Latent Dirichlet Allocation algorithm for topic modeling.

**Results:**

There were 5,781 presentations at MLA annual meetings from 2001–2019. Author disambiguation resulted in approximately 5,680 unique authors. One thousand ninety-three records included a hospital-related keyword in the author field, and 4,517 records included an academic-related keyword. There were 438 presentations with at least 1 international author. The topic model identified 16 topics in the MLA abstract corpus: events, electronic resources, publications, evidence-based practice, collections, academic instruction, librarian roles and relationships, technical systems, special collections, general instruction, literature searching, surveys, research support, community outreach, patient education, and library services.

**Conclusions:**

Academic librarians presented more frequently than hospital librarians, though more research should be done to determine if this discrepancy was disproportionate to hospital librarians' representation in MLA. Geographic affiliation was concentrated in the United States and appeared to be related to population density. Health sciences librarians in the early twenty-first century are spending more time at MLA annual meetings talking about communities, relationships, and visible services, and less time talking about library collections and operations. Further research will be needed to boost the participation of underrepresented members.

## INTRODUCTION

Professional conferences enable librarians and library staff to share projects, trends, resources, and ideas; network with their colleagues; and build relationships. Librarians consider conferences a valuable use of their time and an opportunity for “professional rejuvenation” [1]. The Medical Library Association (MLA) annual meeting gathers together thousands of health information professionals to create presentations, posters, lightning talks, and special content sessions; to meet with their caucus communities; and to receive updates from the association, the National Library of Medicine, and publishers and vendors.

MLA also has a peer-reviewed journal, the *Journal of the Medical Library Association (JMLA)*, previously known as the *Bulletin of the Medical Library Association (BMLA)*. *JMLA* and other journals serve as platforms for professional discourse and preserve the scholarship of the profession. However, research suggests that librarians are more likely to present at a conference than publish a peer-reviewed article [2, 3]. Librarians receive regular calls for submissions from local and national conferences, and each MLA annual meeting needs hundreds of presentations for several days' worth of meeting content. Additional incentives to present may come from a librarian's institution. Some institutions do not support conference attendance unless the attendee is presenting [4], and many institutions require professional activity such as publications or conference presentation for tenure and promotion [5]. The publication process can be slow, so conference presentations may be seen as a more attainable professional activity for a librarian's curriculum vitae (CV).

The Janet Doe lecture, presented by Mark E. Funk, AHIP, FMLA, at the 2012 MLA annual meeting, analyzed the content of *BMLA* and *JMLA* articles to identify key topics from 1961–2010 [6]. He found that the articles described many major changes of the twentieth century: the move from physical to digital information, the expansion of medicine into a broader conception of health care, the separation of librarian services from print collections, a growing emphasis on teaching, and an increase in librarian-led research. No comparable analysis has yet been undertaken for the greater volume of content in MLA annual meeting abstracts, a valuable resource for understanding topics that are currently important to a wide range of health sciences librarians.

Knowing what librarians talk about at MLA annual meetings is important because what we talk about is what we are doing, and what we are doing is creating the future work environments, services, and relationships that will shape and define libraries. Knowing whose voices are heard at annual meetings is important because MLA has identified diversity and inclusion as a top priority [7]. Further research will be needed to identify underrepresented members and create more inclusive meetings. This study seeks to gain initial insight into what is talked about and whose voices are heard at MLA annual meetings and to provide a starting point for larger questions about how to shape future meetings to best reflect MLA's members and priorities.

## METHODS

The purpose of this study was to use computational methods to describe the MLA annual meeting abstracts by analyzing the number of presentations, presentation dates, author information, and abstract content. Meeting abstracts were downloaded from the MLA website, which had portable document format (PDF) versions of meeting programs available for the years 2001 to 2018 [8]. Abstracts for 2019 were downloaded from the 2019 annual meeting website [9]. The PDF files were converted to text format using PDFMiner [10] and then transformed into comma-separated values (CSV) format using Notepad++ [11].

After conversion to text format, most of the meeting files contained a mix of line breaks, semicolons, and numbering to separate the presentations. Structured fields were created from this unstructured data through manually modifying meeting information, such as removing dates and locations, and by using regular expressions to extract presentation information and create consistent line breaks. Semicolons in the unstructured data were consistently used to separate each author's information, but the provided information varied between authors and was not consistently ordered within the semicolon separators. As an example, a record's author section might look like:

Author name, job title, institution name, institution city, institution state;

Author name, library name, institution name, institution city;

Author name, job title, department, institution name, institution city and state

After data cleaning, the meeting programs contained the following fields: Title, Author (including affiliation), Abstract, Type (poster or paper), Year, and uniform resource locator (URL), links to the PDF version of the meeting program. The complete CSV file is available to browse and search online [12].

### Descriptive analysis

Basic descriptive analysis was done using Python pandas [13]. Author analysis included a process to disambiguate authors with minor variations in published names. The Python FuzzyWuzzy package [14] was used to normalize author names within a 90% similarity match. This normalization accounted for small variations, for example, middle initials or typos in the meeting record, but was not able to identify all possible circumstances for name variation from the same individual, such as major name changes or the addition of a full middle name.

Institutional names were not normalized due to the highly variable representation of information in the author column. Inconsistent ordering and punctuation in the descriptions of job titles, institution names, library or department names, and geographic locations, as described above, prevented straightforward automation of a standardized institution field. Since it was beyond the scope of this study to manually extract and reconcile accurate institutional affiliations for each author with authority files, records were instead searched for common keywords related to institution type. For academic institutions, the Author field was searched for the following keywords: *university, school, college*, and *department*. For hospitals, the following keywords were used: *hospital, health system, clinic, health center*, and *medical center*. These searches were not mutually exclusive. Some abstracts had hospital librarians presenting alongside academic librarians, while other abstracts contained affiliation information such as “University Medical Center.”

For each abstract, the first occurrence of any of the academic-related keywords added the record to the academic authorship results, and the first occurrence of any of the hospital-related keywords added the record to the hospital authorship results. Records that contained keywords in the author field from both the academic- and hospital-related keyword lists were counted in both result sets, so an author from “University Medical Center” would be matched for the word *university* and counted in the academic result set and would be matched on the phrase *medical center* and counted in the hospital result set. Identifying and categorizing other institution types was not attempted in this study due to a lack of standard descriptive keywords.

Geographic affiliation was not consistently described in the abstracts. Author fields did not commonly indicate “United States,” “U.S.,” or “USA,” and more often they simply listed the US state of the institution. To determine geographic affiliation, a list of all the US states and territories, as well as state abbreviations, was compared to the Author field to extract the first occurrence of any state name. The resulting list counted the instances of each state name occurring at least once in each Author field. For example, a record containing three authors from Georgia and one author from Florida would be counted once for Georgia and once for Florida. A map was generated using Plotly's choropleth maps library [15] and can be viewed interactively online [16].

A similar approach was used for counting international contributions to MLA meetings. A list of all countries was compared to the Author field to extract the first occurrence of any country name in any Author field. These results were manually reviewed to ensure that personal names or American place names were not included. After double checking that no genuine records existed, the following countries were removed from the list: Georgia, Jamaica, Jordan, Lebanon, and Monaco. As with the institutional and US state search, these results were not mutually exclusive, so international authors who coauthored with each other had their presentations counted once for each represented country.

The descriptive analysis code is available online in a Jupyter notebook [17].

### Automated content analysis

The abstracts were analyzed using topic modeling. Topic modeling is a method of computationally identifying topics in texts. It is an unsupervised approach, not requiring predetermined lists of categories, which is useful for initial exploration of textual data. For this analysis, the topic modeling algorithm chosen was Latent Dirichlet Allocation (LDA) [18]. In simple terms, LDA looks for the distribution of topics in documents (in this case, one document is one abstract) and the distribution of words in the topics (the words in the abstracts that are likely to be associated with the topics). LDA was chosen because of its ubiquity in topic modeling, previous usage in abstract analysis [19], and availability in common Python packages.

After importation, data were cleaned and preprocessed. The data preprocessing was done with the Python libraries spacy [20], NLTK [21], and Gensim [22]. LDA requires both a dictionary, the list of words found in the abstracts and their appearance frequencies in each abstract, and a corpus, the whole collection of processed abstracts to be analyzed. The topic modeling was performed using the Mallet wrapper for Gensim [23]. Mallet is a Java program, but the Gensim wrapper allows Mallet's LDA implementation to be used in Python. After testing Gensim's built-in LDA versus Mallet's LDA, the author considered Mallet to have generated clearer, more intuitive topics from this corpus and proceeded with Mallet. The additional parameters required for LDA are the chunk size, which is the number of documents to be given to the model in each training chunk (set at 200 to very roughly approximate the number of abstracts in any given meeting), the number of passes over the corpus (set to 10), and a random seed option to facilitate reproducibility. Other settings were left as default. To choose the number of topics, test models were generated, and the coherence score was calculated. The coherence score of a topic model assesses the semantic similarity of words in the topic model [24]. Models with 1–20 topics were tested, and the highest coherence score (0.459) was found at 16 topics.

The topics were visualized using pyLDAvis [25]. This visualization can be viewed online [26]. The topic modeling analysis code is available online in a Jupyter notebook [17].

## RESULTS

### Descriptive analysis

#### Overview.

**Figure 1:**
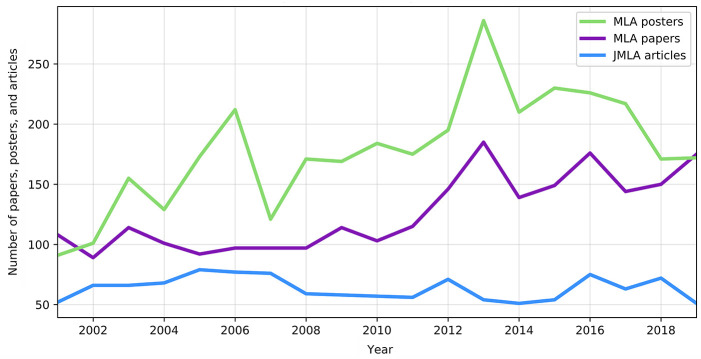
Number of Medical Library Association (MLA) annual meeting presentations and *Journal of the Medical Library Association (JMLA)* articles, 2001–2019

Presentations over time are shown in Figure 1. There were 5,781 presentations at MLA annual meetings between 2001 and 2019. There were 3,388 total poster presentations and 2,393 total paper presentations during this time period, with an average of 304 presentations per annual meeting. The year with the most meeting content was 2013, with 471 total presentations. The first year in the analysis, 2001, and the final year in the analysis, 2019, were the only 2 years in which paper presentations outnumbered poster presentations. As a comparison, 1,205 total items, including non-articles, were published in *JMLA* from January 2001–July 2019, an average of 65 items per year.

#### Authors.

Fuzzy matching was used to disambiguate an original count of 6,732 author names, as described in the methods section, which resulted in approximately 5,680 unique authors. The most prolific author presented 49 times during this 19-year period. There were 28 authors who had 19 or more presentations, averaging at least 1 presentation per year. The top 1% (57 out of 5,680) of authors' names appeared 1,195 times; however, many of these top-presenting authors copresented with other top-presenting authors, so this number does not reflect 1,195 separate presentations.

The most collaborative presentation had 26 authors. Two thousand one hundred ninety-three presentations (almost 38%) had just 1 author, 882 presentations (15%) had 2 authors, and 1,011 (17%) had 3 authors; 1,695 presentations (29%) had between 3 and 26 authors.

**Figure 2:**
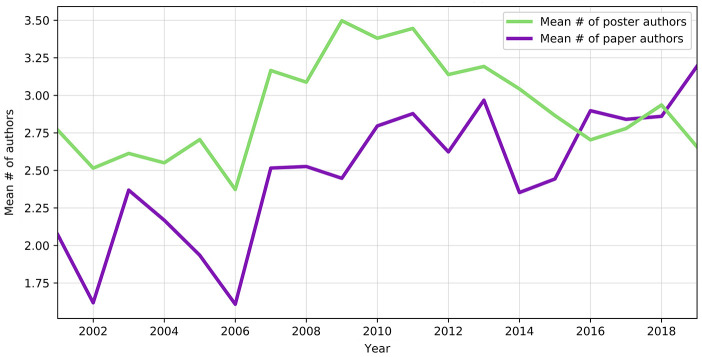
Mean number of authors per presentation type, 2001–2019

Figure 2 shows the mean number of authors by presentation type. The mean number of authors per paper presentation ranged from a low of 1.6 in 2001 to a high of nearly 3.2 in 2019. The mean number of authors per poster presentation ranged from a low of nearly 2.4 in 2006 to a high of nearly 3.5 in 2009.

#### Institutions.

**Figure 3:**
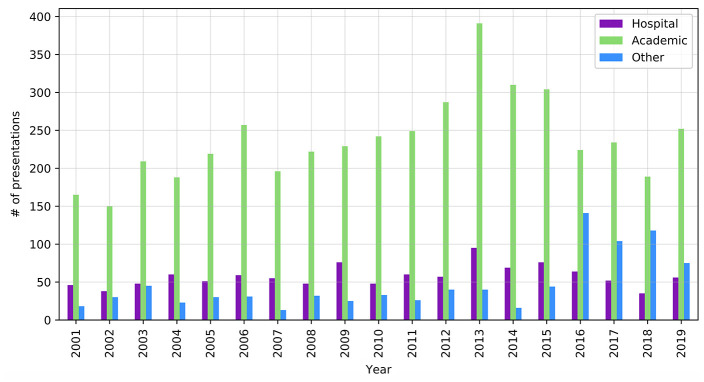
Hospital and academic authorship, 2001–2019

One thousand ninety-three records included a hospital-related keyword in the Author field, which might suggest that hospital librarians authored or coauthored approximately 19% of the total content. An academic-related keyword was included in the Author field for 4,517 records, indicating that academic librarians authored or coauthored approximately 78% of MLA annual meeting content over this 19-year period. There were 888 records that did not include either an academic-related keyword or a hospital-related keyword. These 888 records represented government, public, and special libraries as well as unique or abbreviated names of academic or hospital libraries. For example, “UCLA” would not be captured in the academic result set, although records that contained “University of California, Los Angeles,” would have been captured. Figure 3 displays authorship by institution type. Author fields that did not include an academic or hospital-related keyword are plotted as “other.”

#### US affiliations.

**Figure 4:**
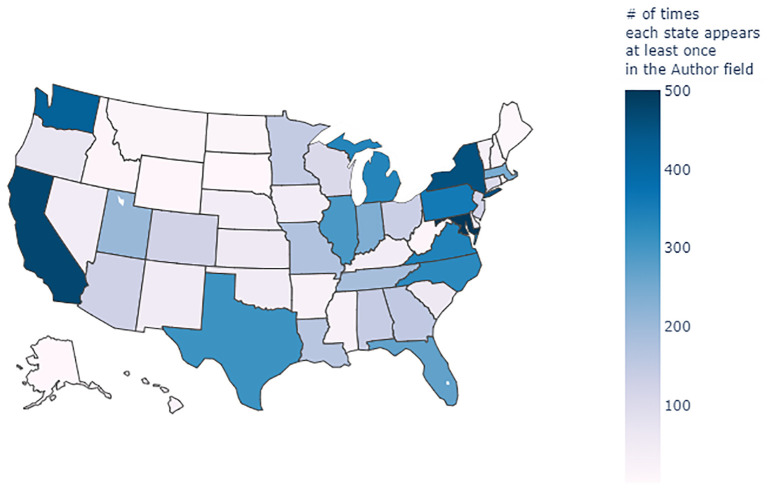
US-affiliated authors, 2001–2019

US states appeared 7,420 times in the dataset, indicating numerous collaborations between institutions in different states. The state that most often appeared at least once per record was Maryland, appearing in 501 records, followed by California, New York, Washington, Pennsylvania, Virginia, Michigan, North Carolina, Texas, Illinois, and Florida as the 10 most frequently appearing states. The only US territory that appeared was Guam, listed on 3 records. An interactive map of the results can be viewed online [16]. Figure 4 shows a static version of the interactive map.

#### International affiliations.

There were 438 presentations with at least 1 international author. Canada was the country that was most frequently represented, with 193 presentations. There were 85 presentations with authors from the United Kingdom, 38 from Taiwan, 22 from China, 22 from Japan, 19 from Australia, 11 from Nigeria, and 10 from the Netherlands. The countries with fewer than 10 presentations were: India (9 presentations); Israel (8 presentations); Qatar (7 presentations); Belgium, Finland, France, Mexico, Switzerland, and Zimbabwe (5 presentations each); Botswana and South Africa (4 presentations each); Brazil, Ireland, and Uganda (3 presentations each); Kenya, Malaysia, Norway, Romania, and Zambia (2 presentations each); and Antigua, Armenia, Costa Rica, Dominica, Estonia, Ethiopia, Germany, Guatemala, Italy, Latvia, Madagascar, Malawi, Mali, Mozambique, Panama, Rwanda, Spain, Turkey, and the United Arab Emirates (1 presentation each).

## AUTOMATED CONTENT ANALYSIS

### 

#### Topics.

The goal of the automated content analysis was to programmatically group keywords in the corpus of the past nineteen years of MLA annual meeting abstracts. The topic model generated groups of word lists, or “topics,” enabling the author to assign a category to each topic based on its highly relevant keywords. The LDA topic model's sixteen categories are identified in the following list. The order reflects the ordering in pyLDAvis' output [26].

The words in parentheses that follow the topic names are the first five words in each topic from the topic model. Italicized words in the descriptions also come from the topic model.

1. **Events** *(member, group, promote, activity, meeting):* This topic seemed to represent a variety of interpersonal events and the *communication* that went into successful events. It showed librarian involvement in professional organizations with terms like *MLA* and *conference*, as well as involvement in broader *communities*. Librarians *planned, marketed,* and *hosted* these events.
2. **Electronic resources** *(resource, user, web, tool, website):* Terms like *portal, interface,* and *software* appeared to describe platforms, while *create, develop,* and *design* might imply librarian creative control or influence. Terms that represented educational aspects, like *tutorial* and *guide,* also appeared.
3. **Publications** *(journal, article, author, publication, citation):* This topic coherently described librarians' responsibility for journal publications and might indicate librarian research as well as scholarly communications support. *Open* and *access* appeared here. Bibliometric studies might also be represented with words like *impact, analysis, compare,* and *report.*
4. **Evidence-based practice** *(clinical, base, evidence, question, practice):* This topic clearly represented evidence-based practice, with librarians *providing* and *presenting* on information resources in a clinical setting *(clinician, physician, resident, case, round).* Terms like *quality, competency,* and *evaluate* seemed to indicate outcomes assessment following evidence-based practice interventions.
5. **Collections** *(library, collection, book, print, electronic):* Unlike topic #3, which appeared to specifically describe librarians' interest in journals, this topic represented collection management. *Usage* was a highly relevant term here, measured by the keywords *change, number,* and *increase. Purchase* and *cost* also appeared.
6. **Academic instruction** *(student, faculty, medical, school, year):* This topic represented *instruction, teaching,* and *learning* in a specifically academic context, evidenced by terms like *college, university,* and *program.* Librarians helped learners *develop literacy* and *skills,* fulfilling *competencies* and enabling them to complete their *assignments.*
7. **Librarian roles and relationships** *(librarian, program, nursing, role, professional):* This topic showed how librarians perceived their professional contexts. *Hospital* and *academic* appeared here as workplace descriptions. *Nurse* and *nursing* seemed to represent nurses as common partners of health sciences librarians. *Liaison* described many librarians' *relationship* with their communities. Librarians brought *knowledge* and *experience* to their community *collaborations,* including through *committee participation.*
8. **Technical systems** *(system, access, process, request, electronic):* This topic described *technical* and *software issues* or *problems,* with librarians seeking to *implement solutions* and *improvements. Staff, manage, management,* and *department* might describe personnel. Library-specific systems like *catalog, database,* and *electronic resource* also appeared.
9. **Special collections** *(medical, project, medicine, digital, material): History, historical, archive,* and *unique* appeared to represent special collections and efforts to *document* those collections digitally via *description* and *metadata. Exhibit* also indicated sharing initiatives. There were also terms that seemed to show large-scale concern for the value of medical special collections, such as *national, world,* and *future.*
10. **General instruction** *(class, session, evaluation, training, online):* Terms like *module* and *workshop* indicated that this topic described instruction more broadly than the academic instruction of topic #6 and might point frequently to an *online* mode of delivery. There was an emphasis on instructional *design* and *assessment,* with terms like *feedback, evaluate, develop,* and *improve.*
11. **Literature searching** *(search, review, database, literature, strategy):* This topic reflected expert searching with *guideline* and *systematic reviews,* as well as references to specific sources like *MEDLINE* and *PubMed.* Librarians *conducted* and *performed* searches, *retrieved* search results, and *studied* the search process.
12. **Surveys** *(datum, survey, study, identify, analysis):* Surveys and questionnaires, common library *research* methodologies, were highly relevant to this topic. The topic also included other *qualitative* approaches such as *interviews*. Terms like *measure, assess,* and *determine* implied motivation for librarian-led research.
13. **Research support** *(research, support, researcher, team, project):* This topic appeared to include research data *management* and *bioinformatics* services. It described *collaborative institutional* partnerships, with librarians as *informationists, team* members *working* to secure or *facilitate grants* and *funding.*
14. **Community outreach** *(health, information, public, community, project):* Terms like *community, local, state,* and *national* highlighted the geography of *outreach*, while *training, resource,* and *access* pointed to specific activities and needs. *NLM, network,* and *region* also appeared, indicating the work of the National Library of Medicine (NLM) and the National Network of Libraries of Medicine (NLM).
15. **Patient education** *(information, health, patient, care, hospital):* This topic seemed to describe information provision to patients, with librarians facilitating *consumer health literacy.* It also might describe patient education information intended for a *physician* and *provider* audience. *Family, child, senior,* and *cancer* indicated health information needs of specific populations.
16. **Library services** *(library, service, staff, science, academic):* This topic related to libraries' *mission* to *serve* their *users, patrons,* and *customers* by *providing space* and *services,* including changing *models* of *reference* services (such as *virtual* reference).

#### Dominant topics.

LDA's output is a list of fractions per abstract. Each fraction in the list represents 1 topic and the level of “aboutness” that the topic contributed to each abstract. Each topic contributes a percentage of content to each abstract, with the total amount of content being 100%.

**Figure 5:**
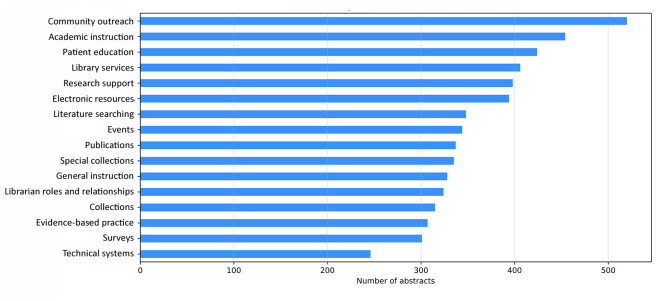
Dominant topics in MLA abstracts, 2001–2019

Abstracts were classified according to which topic contributed the highest percentage to each. The topic that appeared most often as the most dominant topic was community outreach. Figure 5 details the dominance of each topic.

#### Topics over time.

**Figure 6:**
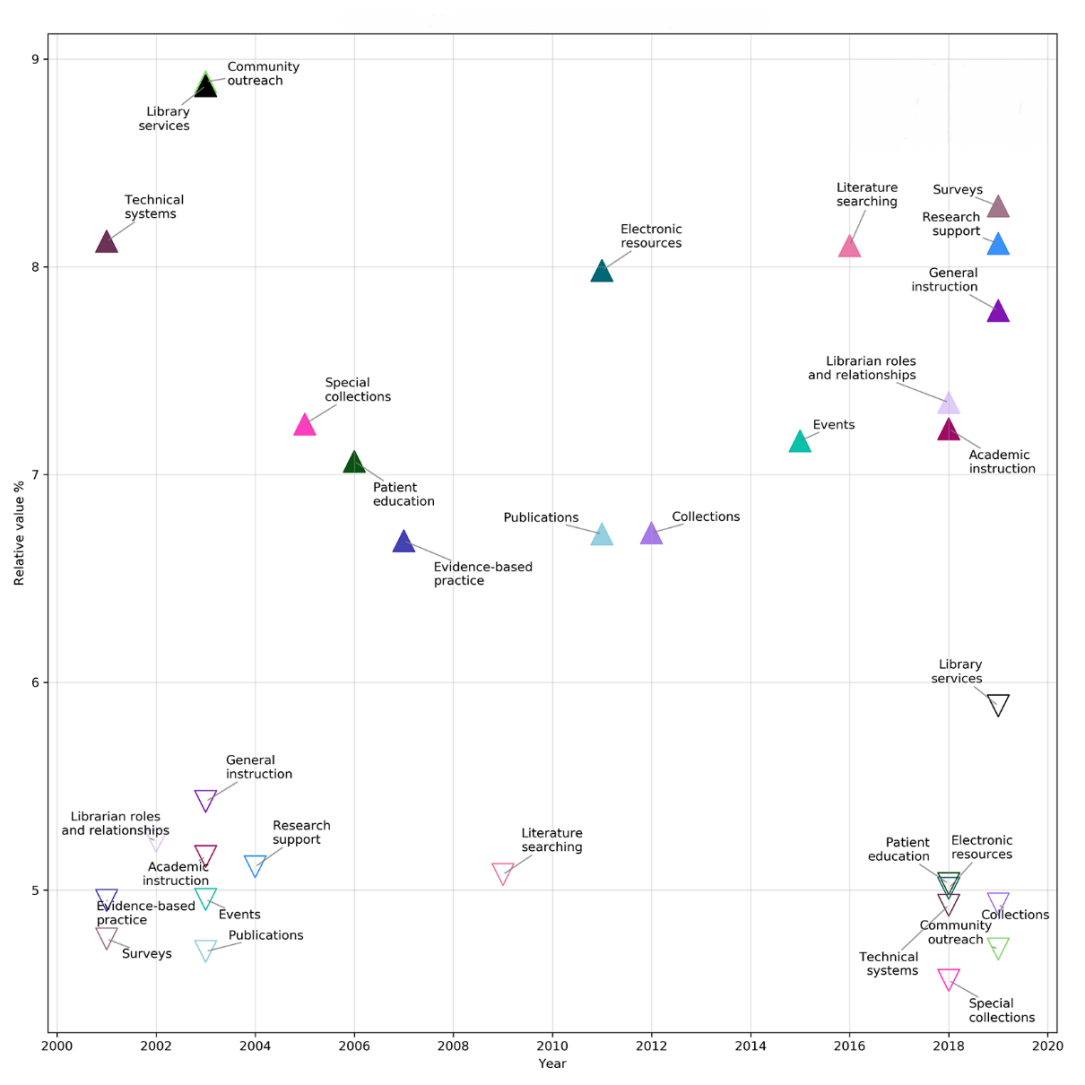
Highest and lowest relative value per topic, 2001–2019

The 16 topics were normalized so that each year's total amount of topic content added up to 100%. Normalization enables insight into how much discussion around each topic happened per year, regardless of the number of abstracts presented that year. For example, the research support topic's total percentage of contribution (i.e., the total sum of the fractional amount of contribution that the research support topic contributed to all abstracts) in the year 2002 was 10.4%, and in 2013, the sum of research support's total contribution had risen to 33.8%. However, 2013 happened to be the meeting with the highest number of total presentations in this 19-year period, with 471 abstracts, so this contribution percentage might not be representative of the proportional discussion of the research support topic in other years with fewer total abstracts. After normalization, research support had its largest proportionate appearance in 2019, with 8.1% of the abstract content, and its lowest proportionate appearance was in 2004, in which its proportional representation was only 5.1%. The minimum and maximum normalized results for each topic are shown in Figure 6.

Solid triangles represent the maximum relative result.

Open triangles represent the minimum relative result.

## DISCUSSION

### Descriptive analysis

The number of paper presentations has been rising since 2017, with a corresponding decrease in the relative number of poster presentations. In 2019, for the first time since 2001, more papers were presented than posters. This reversal might be related to the submission deadline. Prior to 2019, recent years had a single deadline for both poster and paper submissions [27–29]. In 2019, the call for submissions changed to an earlier deadline in October for paper presentations and a later deadline in January for posters. Acceptance notifications for papers were sent in December, allowing submitters to decide before January if they wanted to resubmit a rejected paper abstract as a poster [30]. A 2004 study found that academic librarians considered poster presentations a valuable professional activity but ranked paper presentations as having more value than posters for their promotion and tenure process [31].

Given the typically higher number of poster presentations compared to papers, submitters may believe that they have a greater chance of having their presentation accepted as a poster, and with only one chance to submit, they might prefer to have their submission accepted as a poster rather than rejected as a paper. In 2019, the ability to submit a paper presentation and still have a later chance to resubmit the same abstract as a poster might have encouraged more total paper submissions. This is a positive development, especially for early career librarians who are building a professional reputation. This analysis found that although MLA has been a venue for thousands of unique presenters, a minority of highly prolific authors' names have appeared with disproportionate frequency. If MLA's annual meeting is to prioritize diverse voices, additional research should be done to identify members who are underrepresented at the annual meeting and study the effects of the submission process and other changes on submitters' demographics. While MLA's Medical Library Education Caucus has encouraged new member participation with the New Voices travel stipend and forum [32], additional initiatives to solicit submissions from early career members or other members who have never presented would further encourage presenter diversity.

In 2019, presentations averaged more than 3 authors per paper. Some annual meeting themes were more explicitly collaborative, for example, “Connections: Bridging the Gaps” in 2008 or “Reflect & Connect” in 2010, which theoretically could have encouraged submission of collaborative projects in particular years. The mean number of authors did reach a high point for posters in 2009, with 3.5 authors per poster, corresponding with a meeting theme of “iFusions.” However, collaborative authorship of paper presentations has been increasing every year since 2014, regardless of meeting theme. This collaboration might reflect the growing scale and complexity of librarians' responsibilities. Information contexts are rapidly changing: with more users [33, 34] and ever-increasing amounts of publications and data to manage [35, 36], many of the challenges that await librarians may best be tackled by internal and external collaborations. The growth of coauthorship may also be a reflection of broader scholarly trends, as the average number of authors has been increasing in biomedical sciences as well as social sciences publications [37, 38].

The number of hospital librarians' presentations at MLA annual meetings have consistently been much lower than that of academic librarians. Lessick et al. found in 2016 that hospital librarians were significantly less likely than their health sciences academic counterparts to engage in research activities, present research at conferences, or write research articles [3]. However, many hospital librarians are very active participants in MLA. The current membership makeup of MLA is 2,650 individuals, and as of 2019, the Hospital Library Caucus had 490 members [39]. If this figure of 18% is taken as a rough estimate of the proportion of hospital librarians in MLA, then the low percentage of hospital librarian presentations throughout the meeting years looks more balanced. In 2019, hospital affiliations appeared on 22% of the content, which is greater than the ratio of Hospital Library Caucus members to total MLA members. Further research is needed to determine the true level of hospital librarians' participation at annual meetings.

In the United States, authorship seems to largely reflect population levels. The mid-Atlantic states display particularly high authorship. This region is currently covered by three MLA chapters—Mid-Atlantic Chapter, New York-New Jersey Chapter, and Philadelphia Regional Chapter—indicating a high concentration of health sciences librarians in those states, so correspondingly high meeting representation may be expected. Participation from states or territories with lower populations can be facilitated by targeted solicitation (e.g., special content sessions for rural health), scholarships for travel assistance, or acceptance of virtual presentations.

Outside the United States, librarian participation remains relatively low. MLA occasionally holds joint meetings with international partners, such as the Canadian Health Libraries Association/Association des bibliothèques de la santé du Canada (CHLA/ABSC). Most of MLA's international authors in the study period come from Canada, likely due to the 2016 joint meeting with CHLA/ABSC and to Canada's geographic proximity to meetings in the United States. After Canada, the United Kingdom has contributed the largest number of the authors to MLA, followed by East Asian countries. There is a long tail of remaining countries in MLA's more than 400 internationally authored abstracts. Funding presents a challenge for international participants, although MLA offers a number of international scholarships to attend the annual meeting, as well as discounted membership rates to members from low-income countries [40]. However, some international librarians may also be prevented from participating due to the visa process, especially if they have not received a notification of acceptance for their proposals by the time they submit their visa applications, which may be several months before the meeting. Accepting virtual presentations would make MLA more inclusive of international presenters.

### Automatic content analysis

The topic results can be approximately grouped into two categories:

- **“Looking out”** reflects the people who are part of librarians' jobs, the communities we connect with, and the knowledge and skills we bring, teach, and develop on their behalf: librarian roles and relationships, events, community outreach, general and academic instruction, patient education, literature searching, evidence-based practice, and research support.
- **“Looking in”** involves the materials and processes that are part of our jobs and the ways we define, improve, and reinvent them: electronic resources, collections, special collections, publications, technical systems, library services, and surveys.

With a few exceptions, the “looking out” group topics have peaked in relevance in the last 4 years. There are 3 exceptions to the recent higher relevance of “looking out” topics. The first is community outreach, which peaked in 2003. As seen in Figure 5, community outreach is the dominant absolute topic in over 500 abstracts, but when taken in relation to the other topics, its contribution to annual meeting content has decreased over the past 19 years. This suggests that other topics have possibly replaced the idea of general outreach with more targeted outreach for librarian-specific services, like instruction or research support.

Another factor may be that, in the early 2000s, NNLM and the Regional Medical Libraries began to focus on consumer health [41]. MLA content from earlier years may reflect that shift to public outreach. Patient education also peaked earlier than most of the other “looking out” topics. Patient education seems to imply a clinical or hospital library environment, and its peak in the mid-2000s lines up with a significant decrease in the number of hospital libraries since that time [42], which may account for its diminishment among MLA topics.

The final exception to the “looking out” topics late peak trend is evidence-based practice, which peaked in 2007. This may be a slightly delayed culmination of the 1990s–2000s evidence-based practice paradigm shift in health care. Librarians have used the emergence of evidence-based practice to promote their expertise in searching for and organizing evidence and educating students and providers on finding and using the best quality evidence [43–45]. Though evidence-based practice remains a fundamental aspect of health sciences librarianship, it may be that MLA discussion has shifted away from broader presentations about the importance of evidence-based practice and more toward specific areas, such as expert literature searching.

The “looking in” topics generally peaked in the early to middle years of the 2001–2019 period. The one exception was the surveys topic, which had its high point in 2019, indicating that librarians see their own research as more important than ever. Health sciences librarian–authored research articles have been increasing in number [46], so it makes sense that discussion of research at MLA would also be increasing. The survey topic included terms that were seemingly related to decision making, like *assess* and *determine,* implying that a primary motivation of librarians who undertook research was to make improvements to systems and services. This aligned with Lessick et al.'s study of health sciences librarians, who considered research to be “very important” for “guidance in evaluating, improving, and initiating new library collections, services, and operations” [3].

However, MLA content related to collections and operations has declined. Technical systems peaked at the beginning of this data set, in 2001. Its terms might imply that the late 1990s and early 2000s were a time of implementing new systems—such as websites, integrated library systems, online public access catalogs, link resolvers, and resource sharing networks—and MLA was used a venue to discuss this technological change. The library services topic also peaked early, in 2003. Its terms implied discussion about physical space and reference service, two traditional library provisions that were forever changed by new forms of digital communication and the shift to online collections. Special collections peaked in 2005, and the other collections-related topics peaked in 2011–2012. Although collection development remains a core activity of librarians and information management is highly valued by our communities [47, 48], there has been relatively less discussion about collections at MLA annual meetings in favor of more service-oriented content.

## CONCLUSION

Health sciences librarians in the early twenty-first century are spending more time at MLA annual meetings talking about “looking out” at their communities, and prioritizing library engagement through their relationships and visible services. This shift toward people-centered librarianship, enabled by technology that uncouples librarians from physical buildings and print collections, has been noted for some time. Funk's 2012 analysis found that over the last forty years of the twentieth century, librarians began exploring embedded methods and new ways of outreach to users, prioritized instruction, and embraced technological change [6]. The 2011 Janet Doe Lecture by T. Scott Plutchak, AHIP, FMLA, emphasized the “great age of librarians,” in which the health sciences library profession moved from an era centered on the *library* as an entity into an era defined by the many skills of *librarians* [49].

This study provides additional perspective from librarians using MLA's annual meeting to discuss our current challenges and shape our profession's journey into a twenty-first century, librarian-defined future. As we develop and utilize our knowledge, skills, and expertise to build new relationships with our communities, update our resources and services, and invent new roles for ourselves in the future of health care, we must ensure that MLA and other meetings and conferences represent diverse voices of librarians doing diverse work.

## References

[1] Vega RD, Connell RS Librarians' attitudes toward conferences: a study. Coll Res Libr. 2007 11;68(6):503–16. DOI: 10.5860/crl.68.6.503.

[2] Hoffmann K, Berg S, Koufogiannakis D Understanding factors that encourage research productivity for academic librarians. Evid Based Libr Inf Pract. 2017 12 30;12(4):102–28.

[3] Lessick S, Perryman C, Billman BL, Alpi KM, De Groote SL, Babin TD Jr. Research engagement of health sciences librarians: a survey of research-related activities and attitudes. J Med Libr Assoc. 2016 4;104(2):166–73. DOI: 10.5195/jmla.2016.68. 27076808 PMC4816469

[4] Neville TM, Henry DB Support for research and service in Florida academic libraries. J Acad Librariansh. 2007 1 1;33(1):76–93.

[5] Smigielski EM, Laning MA, Daniels CM Funding, time, and mentoring: a study of research and publication support practices of ARL member libraries. J Libr Adm. 2014 5 19;54(4):261–76.

[6] Funk ME Our words, our story: a textual analysis of articles published in the Bulletin of the Medical Library Association/Journal of the Medical Library Association from 1961 to 2010. J Med Libr Assoc. 2013 1;101(1):12–20. DOI: 10.3163/1536-5050.101.1.003. 23405042 PMC3543134

[7] Knott TL Diversity and inclusion: top priorities for MLA. Full Speed Ahead [Internet]. 12 12 2016 [cited 13 Nov 2019]. <https://www.mlanet.org/blog/diversity-and-inclusion-top-priorities-for-mla>.

[8] Medical Library Association. Past and future meetings [Internet]. The Association [cited 9 Jul 2019]. <https://www.mlanet.org/page/past-and-future-meetings>.

[9] Medical Library Association. MLA '19 | Elevate [Internet]. The Association [cited 9 Jul 2019]. <https://www.eventscribe.com/2019/MLA/>.

[10] pdfminer/pdfminer.six Python PDF parser: fork with Python 2+3 support [Internet]. pdfminer; 2019 [cited 9 Jul 2019]. <https://github.com/pdfminer/pdfminer.six>.

[11] Notepad++. Home [Internet]. Notepad++ [cited 9 Jul 2019]. <https://notepad-plus-plus.org/>.

[12] Myers B MLA meetings [Internet]. Myers B [cited 9 Jul 2019]. <https://c1acj254.caspio.com/dp/0eba700063895a9807334b8d8134>.

[13] pandas. Python data analysis library [Internet]. pandas [cited 16 Jul 2019]. <https://pandas.pydata.org/>.

[14] SeatGeek FuzzyWuzzy. Fuzzy string matching in Python [Internet]. SeatGeek; 2018 [cited 27 Sep 2018]. <https://github.com/seatgeek/fuzzywuzzy>.

[15] Plotly. Choropleth maps [Internet]. Plotly [cited 13 Nov 2019]. <https://plot.ly/python/choropleth-maps/>.

[16] Myers B US-affiliated authors, 2001–2019 [Internet]. Myers B [cited 15 Nov 2019]. <https://bethanymyers.github.io/mla/mla_authors_by_state.html>.

[17] Myers B bethanymyers/mla [Internet]. Myers B; 2019 [cited 5 Sep 2019]. <https://github.com/bethanymyers/mla>.

[18] Blei DM, Ng AY, Jordan MI Latent dirichlet allocation. J Mach Learn Res. 2003 1;3:993–1022.

[19] Sugimoto CR, Li D, Russell TG, Finlay SC, Ding Y The shifting sands of disciplinary development: analyzing North American library and information science dissertations using latent dirichlet allocation. J Am Soc Inf Sci Technol. 2011 1 1;62(1):185–204.

[20] spaCy. Industrial-strength natural language processing in Python [Internet]. spaCy [cited 17 Sep 2019]. <https://spacy.io/>.

[21] NLTK Project. Natural language toolkit: NLTK 3.4.5 documentation [Internet]. NLTK Project [cited 17 Sep 2019]. <https://www.nltk.org/>.

[22] Řehůřek R gensim: topic modelling for humans [Internet]. Řehůřek R [cited 17 Sep 2019]. <https://radimrehurek.com/gensim/index.html>.

[23] Řehůřek R models.wrappers.ldamallet – latent dirichlet allocation via Mallet [Internet]. Řehůřek R [cited 17 Sep 2019]. <https://radimrehurek.com/gensim/models/wrappers/ldamallet.html>.

[24] Röder M, Both A, Hinneburg A Exploring the space of topic coherence measures. In: Proceedings of the Eighth Association for Computing Machinery (ACM) International Conference on Web Search and Data Mining (WSDM) '15 [Internet] Shanghai, China: ACM Press; 2015 [cited 30 Aug 2019]. p. 399–408. <http://dl.acm.org/citation.cfm?doid=2684822.2685324>.

[25] Mabey B Python library for interactive topic model visualization. port of the R LDAvis package [Internet]. Mabey B; 2019 [cited 30 Aug 2019]. <https://github.com/bmabey/pyLDAvis>.

[26] Myers B MLA pyLDAvis [Internet]. Myers B [cited 5 Sep 2019]. <https://bethanymyers.github.io/mla/mla_topics.html>.

[27] Medical Library Association. Mosaic '16: call for submissions [Internet]. The Association [cited 15 Nov 2019]. <https://www.mlanet.org/page/test-page-415>.

[28] Medical Library Association. MLA '17: papers, special content sessions, posters, and lightning talks [Internet]. The Association [cited 15 Nov 2019]. <https://www.mlanet.org/p/cm/ld/fid=900>.

[29] Medical Library Association. MLA '18 FAQ: papers, posters, lightning talks, and special content sessions [Internet]. The Association [cited 15 Nov 2019]. <https://www.mlanet.org/p/cm/ld/fid=1234>.

[30] Medical Library Association. MLA '19 call for submissions [Internet]. The Association [cited 15 Nov 2019]. <https://www.mlanet.org/page/mla19-call-for-submissions>.

[31] Henry DB, Neville TM Research, publication, and service patterns of Florida academic librarians. J Acad Librariansh. 2004 11 1;30(6):435–51.

[32] Medical Library Association. Medical Library Education Caucus [Internet]. The Association [cited 15 Nov 2019]. <https://www.mlanet.org/p/cm/ld/fid=533>.

[33] Center for Health Workforce Studies. Health care employment projections, 2016–2026: an analysis of Bureau of Labor Statistics projections by setting and by occupation [Internet]. The Center [cited 15 Nov 2019]. <https://www.chwsny.org/our-work/reports-briefs/analysis-of-bureau-of-labor-statistics-projections-by-setting-and-by-occupation/>.

[34] National Center for Education Statistics. Indicators: the condition of education: postsecondary education: programs, courses, and completions: graduate degree fields [Internet]. The Center; 4 2019 [cited 15 Nov 2019]. <https://nces.ed.gov/programs/coe/indicator_ctb.asp>.

[35] Johnson R, Watkinson A, Mabe M The STM report: an overview of scientific and scholarly publishing [Internet]. International Association of Scientific, Technical and Medical Publishers; 10 2018 [cited 27 Mar 2020]. <https://www.stm-assoc.org/2018_10_04_STM_Report_2018.pdf>.

[36] NEJM Catalyst. Big data in healthcare: challenges & promise [Internet]. NEJM Catalyst; 2018 [cited 5 Sep 2019]. <https://catalyst.nejm.org/big-data-healthcare/>.

[37] Henriksen D The rise in co-authorship in the social sciences (1980–2013). Scientometrics. 2016 5 1;107(2):455–76.

[38] National Library of Medicine. Number of authors per MEDLINE®/PubMed® citation [Internet]. The Library [cited 15 Jan 2020]. <https://www.nlm.nih.gov/bsd/authors1.html>.

[39] Medical Library Association. Communities home [Internet]. The Association [cited 8 Nov 2019]. <https://www.mlanet.org/p/co/in/>.

[40] Medical Library Association. International programs [Internet]. The Association [cited 15 Nov 2019]. <https://www.mlanet.org/page/international>.

[41] Speaker SL An historical overview of the National Network of Libraries of Medicine, 1985–2015. J Med Libr Assoc. 2018 4;106(2):162–74. DOI: 10.5195/jmla.2018.297. 29632439 PMC5886499

[42] Harrow A, Marks LA, Schneider D, Lyubechansky A, Aaronson E, Kysh L, Harrington M Hospital library closures and consolidations: a case series. J Med Libr Assoc. 2019 4;107(2):129–36. DOI: 10.5195/jmla.2019.520. 31019381 PMC6466508

[43] McCarthy LH Evidence-based medicine: an opportunity for health sciences librarians. Med Ref Serv Q. 1996 Winter;15(4):63–71. 10.1300/J115V15N04_06 10164470

[44] Klem ML, Weiss PM Evidence-based resources and the role of librarians in developing evidence-based practice curricula. J Prof Nurs. 2005 Nov-Dec;21(6):380–7. 16311234 10.1016/j.profnurs.2005.10.004

[45] Perry GJ, Kronenfeld MR Evidence-based practice: a new paradigm brings new opportunities for health sciences librarians. Med Ref Serv Q. 2005 Winter;24(4):1–16. 10.1300/J115v24n04_01 16203698

[46] Gore SA, Nordberg JM, Palmer LA, Piorun ME Trends in health sciences library and information science research: an analysis of research publications in the Bulletin of the Medical Library Association and Journal of the Medical Library Association from 1991 to 2007. J Med Libr Assoc. 2009 7;97(3):203–11. DOI: 10.3163/1536-5050.97.3.009. 19626146 PMC2706445

[47] Marshall JG, Sollenberger J, Easterby-Gannett S, Morgan LK, Klem ML, Cavanaugh SK, Oliver KB, Thompson CA, Romanosky N, Hunter S The value of library and information services in patient care: results of a multisite study. J Med Libr Assoc. 2013 1;101(1):38–46. DOI: 10.3163/1536-5050.101.1.007. 23418404 PMC3543128

[48] Wolff-Eisenberg C US library survey 2016 [Internet]. Ithaka S+R [cited 15 Nov 2019]. DOI: 10.18665/sr.303066.

[49] Plutchak TS Breaking the barriers of time and space: the dawning of the great age of librarians. J Med Libr Assoc. 2012 1;100(1):10–9. DOI: 10.3163/1536-5050.100.1.004. 22272154 PMC3257492

